# Impacts of variable operating conditions on flux and energy efficiency of air gap membrane distillation for brine management

**DOI:** 10.1038/s41598-026-36621-z

**Published:** 2026-04-06

**Authors:** Essam Sh. Mohamed, Ahmed M. Azzam, Amira T. Mohamed, Abdallah Ragab, Ghada Ahmed, Omar Sheta, Mohamed S. Kamel, Elaf Seif, Tamer El-Batt

**Affiliations:** 1 Institute of Global Health and Human Ecology, The American University in Cairo, New Cairo, Egypt; 2 Environmental Research Department, Theodor Bilharz Research Institute (TBRI), Giza, Egypt; 3 Department of Environmental Engineering, The American University in Cairo, New Cairo, Egypt; 4 Department of Computer Science and Engineering, The American University in Cairo, New Cairo, Egypt

**Keywords:** Brine management, Air gap membrane distillation, Membrane characterization, Thermal energy efficiency, Energy science and technology, Engineering, Environmental sciences

## Abstract

In this research paper, we investigate the efficiency of brine treatment using Air Gap Membrane Distillation (AGMD) under varying operational conditions. The study evaluates the performance of the AGMD by examining key metrics, i.e., flux, Salt Rejection Rate (SRR), and Specific Thermal Energy Consumption (STEC) across different flow rates starting from 0.5 L/min to 3.0 L/min and brine salinities ranging from 45 to 65 g/L. To ensure a comprehensive assessment, the membrane was subjected to Scanning Electron Microscopy (SEM) imaging and Fourier-Transform Infrared Spectroscopy (FTIR), both before and after 72 h of testing, providing insights into morphological changes and fouling. Flux varied between 4.7 and 14.0 kg/h/m^2^, SRR ranged from 80 to 99.9%, and STEC values were lowest at 1.0 L/min. Optimal performance was observed at 2.0 L/min with brine concentration up to 55 g/L, achieving flux of 9.8 kg/h/m^2^, SRR > 98%, and STEC between 28,793 and 46,069 kWh/m^2^. SEM and FTIR analysis further highlight the impact of operational parameters on membrane integrity and durability. Experiments were limited to six-hour runs, sufficient for initial fouling dynamics but not long-term degradation. This study offers valuable contributions to the optimization of AGMD processes, aiming to enhance the efficiency and sustainability of desalination technologies.

## Introduction

The rapid change in population growth and economic development has significantly affected water consumption and enhanced fresh water reserves^1,2^. This depletion paved the road for a development in desalination technologies to secure fresh water from saline sources^3,4^. The driving forces that separate the ions from water are what classify the desalination technologies^5^. There are three common desalination technology categories: reverse osmosis (RO), electrodialysis (ED), and evaporation technique using multi-effect distillation (MED). However, conventional desalination methods produce concentrated brine with salinity levels exceeding 70 g/kg. Also, freshwater recovery rates are low, resulting in the production of large volumes of concentrated brine exceeding 50% of freshwater production that require proper management^6^.

Thus, membrane distillation (MD) proved to be an advanced method for managing highly concentrated solutions. The distillation process can be carried out using various techniques; the most used ones are air-gap membrane distillation (AGMD), direct contact membrane distillation (DCMD), vacuum membrane distillation, and swept gas membrane distillation^7,8^.

Recently, AGMD has started to become a promising desalination technology, presenting a viable solution to the ever-growing global freshwater scarcity. AGMD is a thermally driven membrane technique that utilizes differences in the pressure of a vapour to transport water vapour through a hydrophobic microporous membrane, thus separating salts and impurities from the feed solution. Unlike previous AGMD studies focused primarily on seawater desalination, this work specifically addresses brine management under high salinity conditions (45–65 g/L), providing new insights into flux–energy trade-offs and fouling dynamics. The key principle lies in the creation of a vapour gap between the membrane and the feed brine; this allows feed molecules to evaporate and subsequently condense on the side of the membrane, leaving behind salts and contaminants^9,10^.

A conventional one-stage AGMD can work at an efficiency of around 4 to 8%. To be able to recover with an efficiency of 70% one of two methods can be used: batch recirculation or concentration stages. When using the batch recirculation method, brine is kept in a circulation mode until we achieve the desired water recovery. A downside of this method is that it is less effective in the case of continuous operation. When using concentration stages, several AGMD modules are set downstream of each other, this method allows for a more predictable operation and higher recovery of freshwater^11,12^. Moreover, AGMD requires increasing water flow to decrease the active area of the used membrane to reduce the related costs of capital. However, this necessity reduces its energy efficiency and elevates operating expenses^13^.

Several studies have been conducted to assess the impact of various parameters for operation on the improvement of the performance and effectiveness of AGMD. To evaluate the efficiency and effectiveness of AGMD, several critical measurements and parameters come into play. The performance of AGMD is examined concerning permeate flux, energy utilization, temperature, and brine concentration. Permeate Flux, denoted in kg/m^2^/h, measures the amount that passes from the active area of the membrane over a defined area and time, providing insights into the membrane’s efficiency. Energy utilization can be assessed through several measurements, such as the Gained Output Ratio (GOR), which provides a clear measure of the process efficiency by computing the ratio of produced freshwater vapour to the input energy, and the thermal efficiency (η). Monitoring temperature and concentration polarization, furthermore, requires observing how the temperature changes across the surface of the membrane and how solute concentration in the feed water affects vapor efficiency, transport, and overall performance. These parameters serve as the key measurements to assess how different operating conditions influence the capacity of the desalination operation^14,15^.

Key metrics for evaluating the implementation of pilot-scale techniques of thermal desalination include specific electrical energy consumption (SEEC) and specific thermal energy consumption (STEC). Higher energy usage leads to increased system costs. The techno-economic model of MD systems demonstrates that one of the main factors affecting the cost of the operation is the high amount of thermal energy. Basically, STEC is inversely proportional to the production of distillate^16,17^.

As for this study, the objective is to detect the impact of various parameters (Brine concentration and flow rate) on the permeate flux, permeate total dissolved solids (TDSp), and salt rejection ratio (SRR) in the AGMD process as well as the membrane characteristics. By varying the feed parameters, we seek to evaluate their impact on the water permeation rate through the membrane. In addition to assessing the permeate flux, at constant ∆T (40 ± 2 °C) between hot feed water (70 ± 2 °C) and cooling one (30 ± 2 °C) using polytetrafluoroethylene (PTFE) membrane (0.2 μm). Also, we aim to analyze changes or fouling effects on the membrane during the operation, where Scanning Electron Microscopy (SEM) and Fourier-Transform Infrared Spectroscopy (FTIR) were utilized to analyze the configuration and chemistry of the membrane. These analysis techniques allow us to examine any structural changes or fouling effects during operation. By correlating feed parameters with membrane characteristics and permeate flux, we can better understand the interplay between feed conditions and membrane behavior. Thus, this work seeks to supply insights into the effect of the operation conditions, in addition to the impact on the durability and fouling tendency of the used membrane.

## Materials and methods

### Description of the unit

**Fig. 1:**
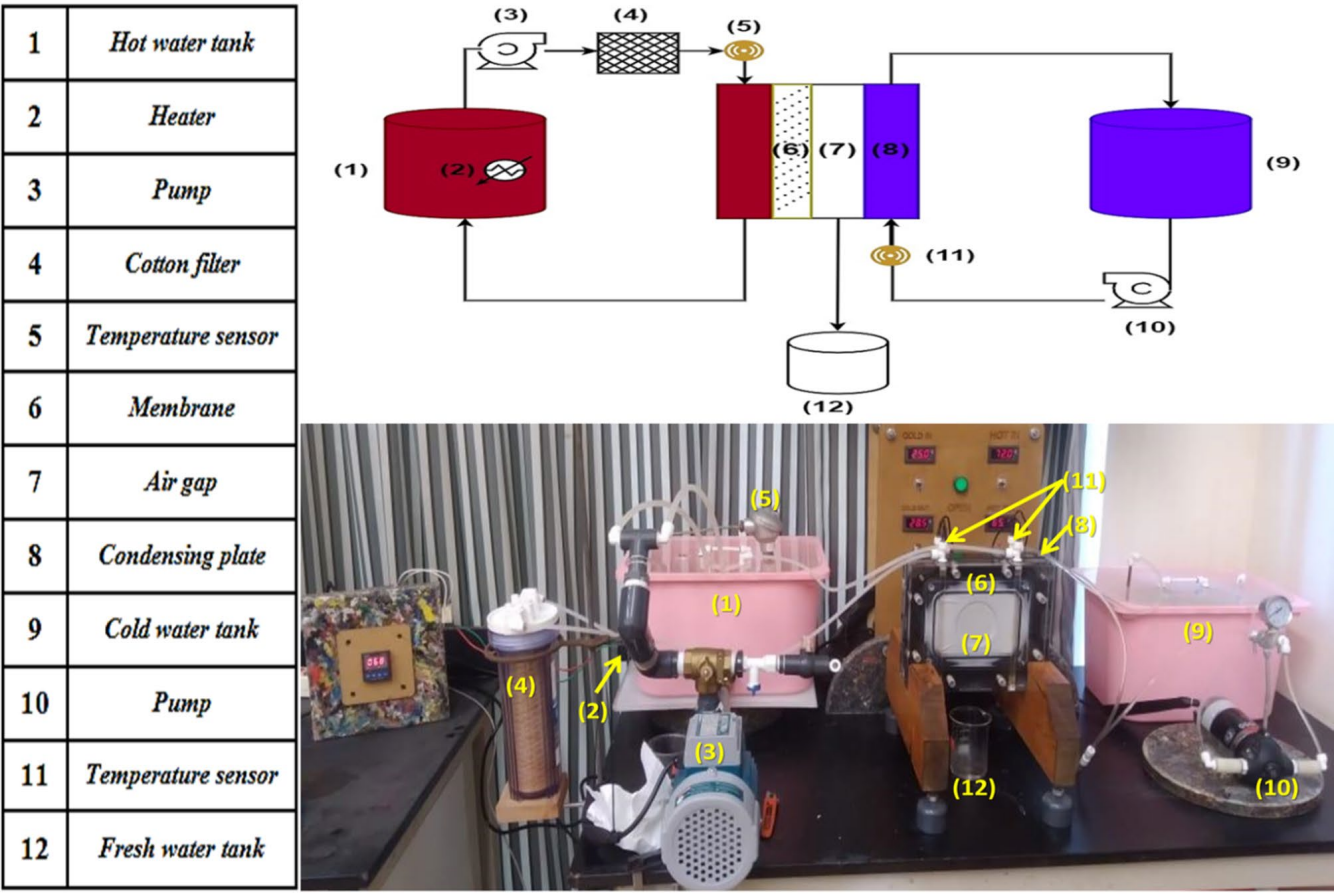
Schematic diagram of the AGMD module used in our work.

The experiment utilized an air gap membrane distillation (AGMD) system, one unit (Sterlitech Corporation, USA) operated with an air gap thickness maintained at 5.6 mm for all experiments. The AGMD system comprises a hot water cycle and a cold water cycle, each with dedicated tanks, pumps, and temperature control mechanisms. The recycling hot water cycle includes a feed tank with a capacity of 10 L, a variable flow hot water pump capable of flow rates ranging from 0.5 to 3.0 L/min, and an electric heater to maintain the feed brine at the set temperature. Moreover, the cold water cycle consisted of a feed tank with a capacity of 10 L and a fixed-flow cold water pump with a flow rate of 1.3 L/min, ensuring the operational efficiency of the cooling loop, as shown in Fig. 1.

### AGMD performance estimators

The AGMD module employs hydrophobic microporous PTFE membranes (Sterlitech Corporation, USA), details of which are summarized in Table 1. The system operates by circulating the hot saline feed water via the membrane module of the hot part, while the cold water is circulated via the cold one, creating the necessary temperature gradient to drive vapor through the membrane.

**Table 1:** AGMD module and membrane characteristics.

Parameter	Unit	Value
Membrane material	–	PTFE
Membrane pore size	µm	0.2
Membrane sheet thickness	µm	76–152
Membrane active area	cm^2^	140
Maximum operating pressure	bar	15
Maximum temperature	ºC	88
O-rings	–	Viton
Gaskets	–	Viton
Spacer	–	Acrylic
Condenser plate	–	Aluminum
Air gap thickness	mm	5.6
Feed channel depth	mm	1.9
Feed channel width	mm	146

### Process description

Synthetic saline water was prepared using Sodium Chloride (NaCl) concentrations (45, 55, 65 g/L) and heated using the system’s heater. The hot saline water moves from the feeding tank of hot water into the spread section of the MD unit by the hot water pump. Concurrently, cold water was pumped into the cold chamber of the MD unit. The differential between the temperature of the feed and the cold chambers facilitated the vapor transfer into the air gap, where it condensed on the cold plate of condensing and then assembled as freshwater. The hot and cold water streams were continuously recirculated to maintain steady-state conditions. The experimental setup included a cotton filter, temperature sensors at key points, and a digital interface for monitoring and data logging.

### Experimental design and data collection

The experimental parameters varied including feed brine concentrations and flow rate. The resulting permeate is collected in a flask and weighed to ascertain the volume of freshwater generated. Using a salinity meter, the TDSp is measured to assess the SRR. The experiments were conducted for six continuous hours with permeate samples collected and analyzed every hour using a salinity sensor. The results for each experimental run were represented by their mean values.

Each experiment was repeated three times under identical conditions, and the reported values represent the average of these replicates.

The experiments were conducted for six continuous hours with permeate samples collected and analyzed every hour.

### Membrane characterization

SEM of the model (JEOL, JCM-6000 Plus, Japan) set at a voltage of 15 kV, was used to study the appearance of the used membrane by AGMD compared with a virgin one. To make the samples conductive, we cut different membrane areas and mounted them on the BT-SEM with the aid of carbon tape. Then, we sputter it with a gold layer with the aid of (HUMMER^®^ 8, PN-3807007–220 V-ED-REV-C)^18^. In order to study the chemical bond difference between the used and the virgin membrane, attenuated FTIR was employed using a BRUKER ALPHA II transmission unit, through a range of wavenumber from 400 to 4000 cm^− 1^ and 0.5 cm^− 1^ step size. The spectrometer runs Opus 7.8 software^18^.

### Performance metrics

#### Salt rejection ratio (SRR)

SRR is calculated to evaluate the efficiency of the membrane in removing salts from the feed water. The salt rejection is calculated as follows in Eq. (1):

1 $$\:SRR=\left(1-\frac{{c}_{p}}{{c}_{f}}\right) \times 100$$

#### Specific thermal energy consumption (STEC)

The STEC is a critical parameter for evaluating the efficiency of the AGMD process. It represents the quantity of thermal energy needed to create 1.0 m^3^ of distillate water and is calculated using Eq. (2)^19^:

2 $$\:STEC=\frac{{\dot{\mathrm{Q}}}_{feed}+{\dot{\mathrm{Q}}}_{coolant}}{{\mathrm{V}}_{p}}=\frac{{\dot{\mathrm{m}}}_{f}*{C}_{p,f}*({T}_{f,in}-{T}_{f,out})+{\dot{\mathrm{m}}}_{c*{C}_{p,c}*({T}_{c,out}-{T}_{c,in})}}{{\mathrm{V}}_{p}}\:$$

## Results and discussion

**Fig. 2:**
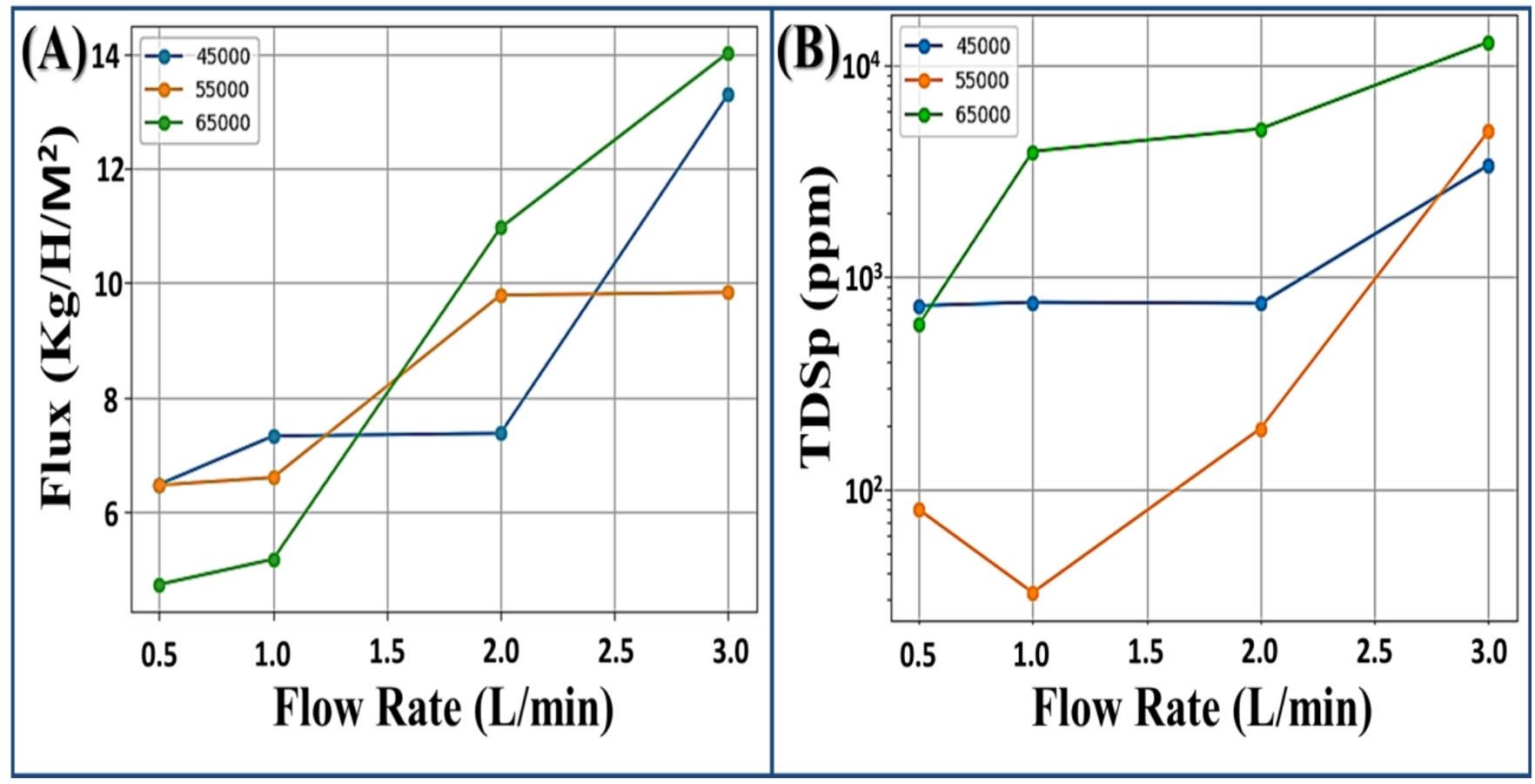
Effect of inlet flow rate on permeates (**a**) flux and (**b**) salinity at salinities 45, 55, and 65 g/L.

Figure 2a showed the impact of different inlet flow rates on the flux with various brine concentrations (45, 55, 65 g/L). The flow rate has direct coloration with flux, where at 0.5 L/min flow rate the flux were 4.7, 6.4, and 6.4 kg/h/m^2^ at 65, 55, and 45 g/L, respectively, then by increasing flow rate the flux increased reaching to 14.0, 13.3, and 9.8 kg/h/m^2^ at 65, 45, and 55 g/L, respectively, at 3.0 L/min flow rate. Flux varied between 4.7 and 14.0 kg/h/m^2^ depending on salinity and flow rate.

Figure 2b, shows the effect of different inlet flow rate on fresh water salinity (TDSp) of different brine concentrations. By increasing flow rates, the fresh water salinity increased, where at 0.5 L/min flow rate, the mean freshwater salinities were 80, 597, and 731 ppm at 55, 65, and 45 g/L, respectively, then by increasing flow rate to 3.0 L/min the freshwater salinities reached 3351, 4840, and 12,766 ppm, respectively.

At high flow rate (3.0 L/min), brine 65 g/L produced high flux (14.02 kg/h/m^2^) but TDSp was high (12766 ppm). At moderate concentration (55 g/L), flux was 9.83 Kg/h/m² with TDSp 4840 ppm.

**Fig. 3:**
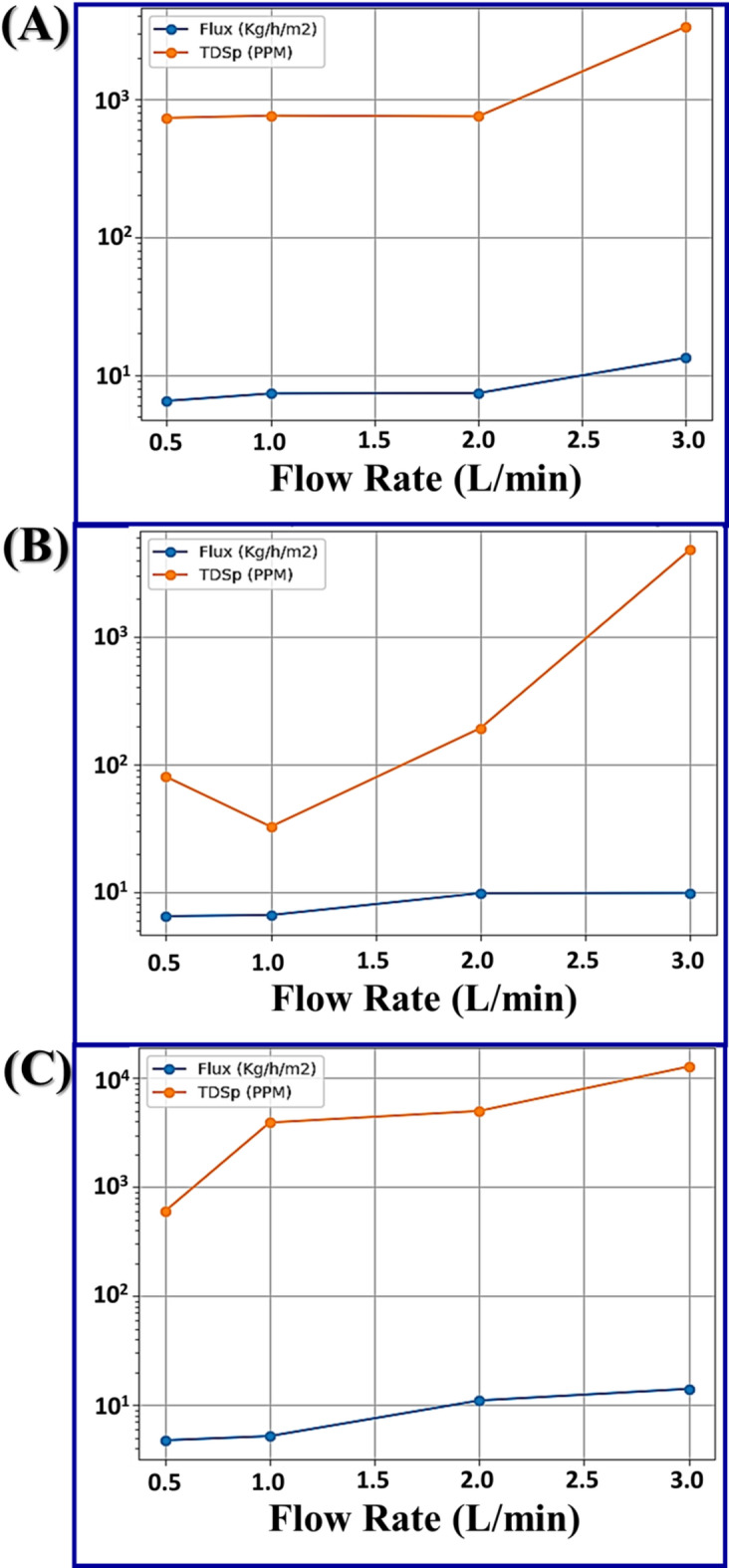
Effect of inlet flow rate on permeates flux and salinity at initial salinity (**a**) 45 g/L, (**b**) 55 g/L, and (**b**) 65 g/L.

From the data, the treated brine concentration 45 g/L showed variations in flux ranging from 6.47 to 13.29 kg/h/m^2^ and freshwater salinity from 731 to 3351 ppm by changing the inlet flow rate from 0.5 to 3.0 L/min Fig. 3a. However, brine concentration 55 g/L represented flux variations from 6.46 to 9.83 kg/h/m^2^ and freshwater salinity from 80 to 4840 ppm Fig. 3b. Meanwhile, the brine concentration 65 g/L recorded variations from 4.72 to 14.02 kg/h/m^2^ and freshwater salinity from 597 to 12,766 ppm Fig. 3c. So, the data cleared that at high flow rate (3.0 L/min), the treated high concentration brine (65 g/L) produced high flux (14.02 kg/h/m^2^), but TDSp was high (12766 ppm), on the other hand treated low concentration brine (45 g/L) produced high flux (13.29 kg/h/m^2^) with low TDSp (3351 ppm). Finally, the treated moderate concentration brine (55 g/L) produced moderate flux (9.83 kg/h/m^2^) with low TDSp (4840 ppm). These outcomes agree with Alsalhy et al.^20^, who investigated the impact of flow rate and concentration on AGMD, where they recorded permeate flux increasing by 30% by increasing the flow rate of input brine from 0.25 to 0.55 L/min, but lowered by 20% as feed brine concentration increased from 0 to 45 g/L.

**Fig. 4:**
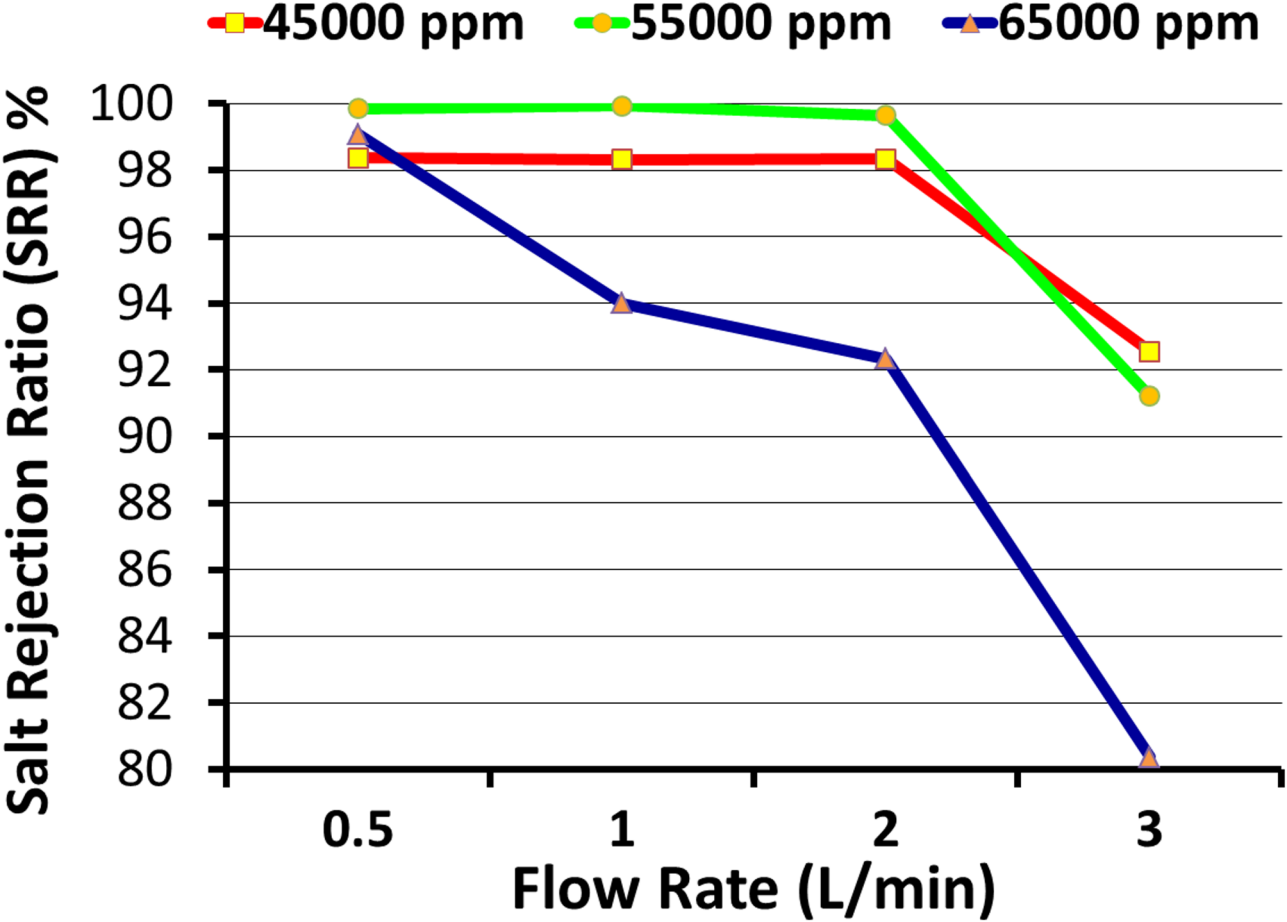
The effect of the flow rate on the salt rejection ratio (%) at different treated brine.

Figure 4 shows the impact of the flow rate of treated brine on the SRR of produced freshwater, where at a flow rate of 0.5 L/min the SRR represented 98.4, 99.9, and 99.1% for 45, 55, and 65 g/L brine, respectively, however by increasing flow rate to 1.0 L/min the SRR reached to 98.3, 99.9, and 94.0%, respectively. Meanwhile, up to 2.0 L/min, the SRR was still more than 98% for brine concentrations of 45 and 55 g/L and more than 92% for brine 65 g/L. On the other hand, by increasing to 3.0 L/min, the SRR decreased to 92.6, 91.2, and 80.4%, respectively. SRR remained > 98% at 2.0 L/min for 45–55 g/L, but decreased to 80% at 65 g/L, 3.0 L/min; therefore, the repeated results of the proposed model and its accuracy make it a model that can be applied practically and industrially^10^.

**Fig. 5:**
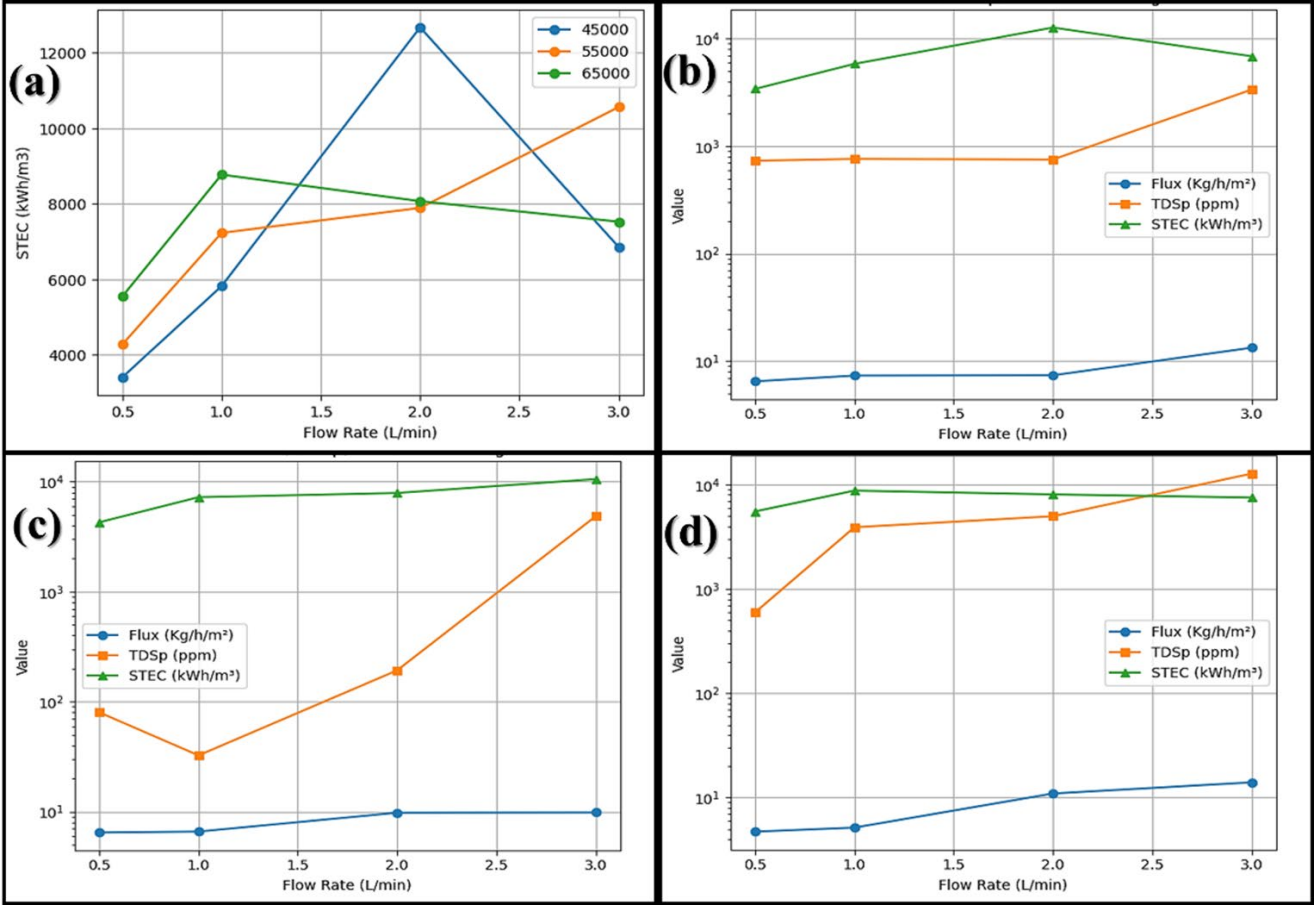
(**a**) The impact of the flow rate on the STEC at different treated brine concentrations; comparison between the effect of the flow rate on the flux (kg/h/m^2^), and TDSp (ppm), and STEC at different treated brine concentrations (**b**) 45 g/L, (**c**) 55 g/L, and (**d**) 65 g/L.

Figure 5 represents the impact of different flow rates on STEC, flux, and TDSp at different concentrations of treated brine. Figure 5a shows big differences and very high STEC values at 0.5 L/min, where STEC recorded 14,320; 39,456; and 53,586 kWh/Kg, for 45, 65, and 55 g/L treated brine, respectively, while at 1.0 L/min, the STEC values were reduced to 19,605 and 25,380 kWh/Kg, for 55 and 65 g/L when using 1.0 L/min, respectively. After that, the STEC values increased to 28,793; 34,564; and 46,069 kWh/kg, for 45, 55, and 65 g/L brine, respectively, at a 2.0 L/min. However, at 3.0 L/min, the STEC values recorded the nearest values 36,413; 39,282; and 40,795 kWh/m^3^, for 45, 55, and 65 g/L brine, respectively. As anticipated, the STEC values measured in this lab-scale study are higher than those reported for commercial or large-scale pilot systems. This is a well-documented characteristic of small-scale thermal desalination units^19,21,29^. On the pilot scale, the STEC values for membrane distillation are significantly lower and vary from 49 to 810 kWh/m^3^^21,22^. Also, several strategies have been experimented with aiming to reduce energy consumption in thermal desalination processes. An experimented strategy has been the development of multi-stage AGMD systems, which are designed to recover and reuse the latent heat of condensation from one stage to preheat the feed in the next, thereby lowering the net thermal energy input per unit of distillate produced^30,23^. The implementation of these strategies is therefore critical to overcoming the persistent technical and economic challenges associated with high-salinity brine desalination^24^.

Even though the thermal energy needed for MD has been well-documented, the electrical consumption has not been as thoroughly explored. Pumping is commonly employed to enhance MD performance, but its impact on energy consumption is often overlooked^22,25^. Our results showed high STEC values; this may be attributed to using one small unit of AGMD repeating units, which leads to a high consumption of energy.

For comparison results of treatment of different brine concentrations at variable flow rates as demonstrated in Fig. 5b–d, data reported that 2.0 L/min flow was the most suitable for treatment of three different brine concentrations, where at this flow rate the flux was accepted and ranged from 7.3 to 10.9 kg/h.m^2^ and the TDSp ranged from 192 to 4986 ppm, while the STEC recorded moderates values between 28,793 and 46,069 kWh/Kg. Other researchers compared the performance of AGMD through varying operating conditions and membrane properties. Varying operating conditions were feed temperature, flow rates, and air gap width. As for the membrane properties, the varied properties were the size of its pore, thickness, porosity, and thermal conductivity. As for the effect of operating parameters and membrane properties on AGMD performance, feed temperature was found to have a powerful influence on energy efficiency and permeate flux. Another factor affecting the AGMD performance is the rate of flow and the thickness of the air gap. On the other hand, membrane porosity and thermal conductivity have no significant impact. Increasing feed temperature from 50 to 80 °C led to a 200% gain in flux, increasing from 4.07 to 12.22 kg/(m^2^ h)^26^. Regarding energy efficiency parameters, raising the temperature feed led to increased GOR from 0.725 to 0.734 and increased energy efficiency from 96.2 to 98.2%. Also, increasing the flow from 0.2 to 8 L/min enhanced flux by 67.19% from 6.38 to 10.68 kg/(m^2^ h). However, thicker air gaps of 5.6 compared to 0.6 mm reduced permeate flux by 36.8%^12^. In addition to permeate flux and GOR, Abu-Zeid et al.^23,27^ computed performance ratios under various application conditions for brine input flow rate and salinity with and without a vacuum pump. Increasing brine input flow showed an increase in flux, while a reduction occurred in all other parameters. Also, increasing feed salt concentration showed a decrease in all performance parameters^23,27,28^. We attribute the increased flux at higher flow rates to the reduction of thermal boundary layer resistance (less temperature polarization), and the subsequent decrease in SRR at the highest flow rate (2.5 L/min) is linked to the increased shear stress, potentially inducing pore wetting or leading to a rapid buildup of crystallizing foulants near the pore entrance due to high local concentration factors^24,25,29,30^.

### Membrane characterization

The measurement values of the laminated hydrophobic PTFE membrane features are summarized in Table 1. The average pore size value is 0.2 μm as stated by the producing company. The bubble point pore size was ≥ 17 in dispersions in isopropyl alcohol (IPA). One of the major key parameters when determining the flux in membrane distillation (MD) is the membrane’s porosity. It represents the available volume that can be used for the vapor movement. While the thickness of the membrane was 76–152 μm, with water input flow pressure > 37 psi. Finally, the membrane clean air flow was 0.26–0.55 L/min cm^2^ at 70 mbar.

**Fig. 6:**
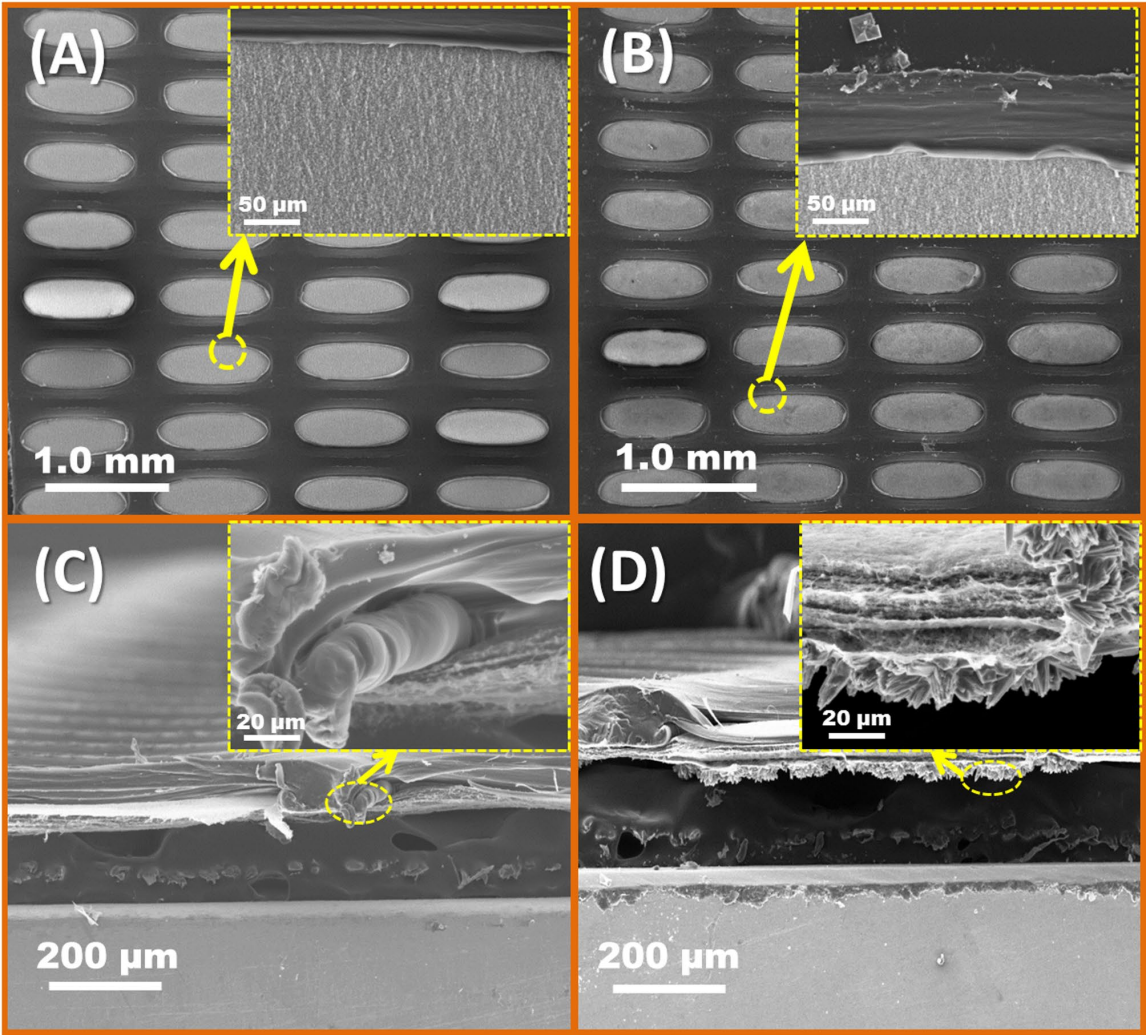
SEM images of PTFE membranes (0.2 µ) surface before (**a**) and after (**b**) cross section and before (**c**) after (**d**) 72 h using at different magnifications.

Figure 6 shows both SEM images of the texture and the cross-section of the membrane before and after use for 72 h. On the unused membrane surface, the nodes were connected by fibers, the nodes and fibers were arranged orderly, the fibers were along the stretching direction, and the fiber spacing was equal. In addition, the nodes showed an ordered microstructure. Moreover, images of the cross-section indicated that the pores in the used membrane were more twisted than the pores in the unused membrane; a crystalline structure could be seen in the surface and cross-section images of the used membrane, with more solidification of the pore section for the used membrane. Sodium chloride crystals were also observed to be trapped in the membrane pores. Elevated concentration of these inorganic elements was thought to enhance membrane wetting and reduce membrane rejection^18,24,29^.

**Fig. 7:**
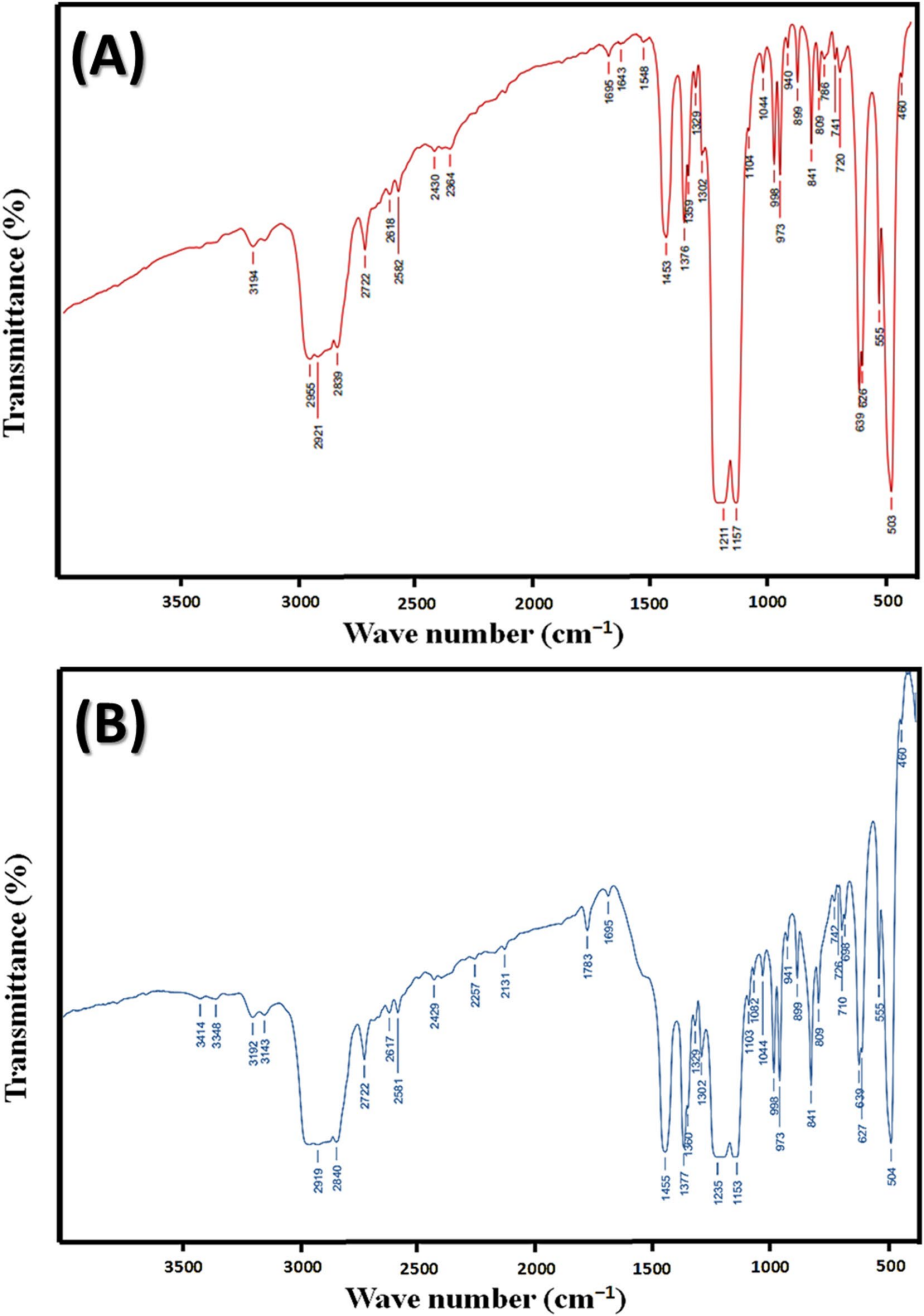
FTIR of PTFE membranes (0.2 µ) (**a**) before and (**b**) after of the 72 h of continuous testing.

To investigate the chemistry of virgin and used membranes, Fig. 7 represents the FTIR analysis of both. The spectra of FTIR of the virgin one revealed ordinary permeability peaks at 1211 cm^− 1^ and 1157 cm^− 1^ attributed to C–F bonds, while the absorption bands observed near 2955 and 2839 cm^− 1^ (Fig. 7a) are equal to C–H coordinated and uncoordinated extension, respectively. The presence of polysaccharides was confirmed by the existence of these bands^25,30^. On the other hand, the used one, has many other peaks at 1082 cm^− 1^, 1235 cm^− 1^, 1695 cm^− 1^, 1783 cm^− 1^, 2257 cm^− 1^, 3348 cm^− 1^ and 3414 cm^− 1^ (Fig. 7b). The band at 1082 cm^− 1^ is specified to stretching vibration of the carbohydrate C–O bonds, while 1783 cm^− 1^ is specified to C–O–C, C–OH and C–C bonds of alicyclic ether. The intensity of the peaks of the used membrane properties is significantly reduced after contamination due to the accumulation of salt crystals^26,31^. Furthermore, Fig. 7b displays an absorption peak near 1235 cm^− 1^, which is linked to the natural oxide layer. Minor organic contamination on the surface is suggested by peaks between 2840 and 2919 cm^− 1^, attributed to C–H absorption. Transmission method results also indicate absorption at approximately 1082 cm^− 1^^27,32^. However, peaks at 3348 cm^− 1^ and 3414 cm^− 1^ are referred to as –OH group expansion pulse; moreover, the methylene (= CH_2_) group is represented at 2840–2919 cm^− 1^^28^.

## Conclusion

Few studies in the literature have focused on brine desalination, with most research concentrating on seawater desalination. Therefore, this paper addresses a critical gap by specifically focusing on brine desalination. Optimal performance was observed at 2.0 L/min with brine concentration up to 55 g/L, achieving flux of 9.8 kg/h/m^2^, SRR > 98%, and STEC between 28,793 and 46,069 kWh/m^2^. The primary aim of this research was to achieve effective brine treatment, addressing the environmental drawbacks of brine discharge. The results indicated significant variations in flux, highlighting the system’s challenges and potential under different operating conditions. Higher flow rates and salinities generally resulted in lower SRR. As for the system’s energy efficiency, the lowest STEC values were observed at an optimal flow rate for all salinities, suggesting a specific operational condition for enhanced energy efficiency. Results show that applying a flow rate of up to 2.0 L/min with brine concentration up to 55 g/L recorded an SRR of more than 98% which balances high permeate flux with excellent Salt Rejection Ratio (SRR) and acceptable Specific Thermal Energy Consumption (STEC). SEM and FTIR analysis provided insights into the morphological changes and fouling of the membrane, underscoring the impact of operational parameters on membrane integrity and durability. It should be noted that the experiments were limited to 6-h runs, which were sufficient for initial fouling and wetting dynamics but not long-term degradation. Future work should focus on scaling AGMD modules, integrating energy recovery strategies, and applying IoT/ML for predictive optimization. This research contributes to the optimization of AGMD processes, aiming to enhance the efficiency and sustainability of desalination technologies. This interdisciplinary approach promises significant improvements in the sustainability and effectiveness of brine management solutions. This study also paves the way for integrating advanced technologies, such as machine learning and IoT, to optimize and control real-life AGMD systems. Future research could explore these technologies to predict and adjust operational parameters in real-time, enhancing system responsiveness, performance, extended run times, upscaling, and energy efficiency. This interdisciplinary approach promises significant improvements in the sustainability and effectiveness of desalination solutions.

## Data Availability

All data generated during this study are included in this published article.

## List of symbols

AGMD: Air gap membrane distillation
MD: Membrane distillation
PTFE: Polytetrafluoroethylene
TDSp: Total dissolved salts of permeate water (ppm)
SRR: Salt rejection ratio (%)
STEC: Specific thermal energy consumption (kWh m^− 3^)
FTIR: Fourier transform infrared spectroscopy
SEM: Scanning electron microscopy

## Symbols

*c*_*p*_: Concentration of NaCl in the produced distillate (g L^− 1^)
*c*_*f*_: Concentration of NaCl in the feed (g L^− 1^)
*Q*_*f*_: The feed flow rate (L S^− 1^)
*ρ*_*f*_: Feed density (kg m^− 3^)
*C*: Specific heat capacity (J kg^− 1^ °C)
*T*_*hot, in*_: Hot inlet temperature (°C)
*T*_*cold, out*_: Cold outlet temperature (°C)
*Jw*: Distillate flux (L/m^2^h)
*A*: Membrane active area (m^2^)
*∆T*: Difference temperature between hot water inlet and cold water inlet (°C)
